# Bactericide, Immunomodulating, and Wound Healing Properties of Transgenic* Kalanchoe pinnata* Synergize with Antimicrobial Peptide Cecropin P1 In Vivo

**DOI:** 10.1155/2017/4645701

**Published:** 2017-02-23

**Authors:** A. A. Lebedeva, N. S. Zakharchenko, E. V. Trubnikova, O. A. Medvedeva, T. V. Kuznetsova, G. A. Masgutova, M. V. Zylkova, Y. I. Buryanov, A. S. Belous

**Affiliations:** ^1^Russian Academy of Sciences, Branch of Shemyakin-Ovchinnikov Institute of Bioorganic Chemistry, Pushchino, Moscow Region, Russia; ^2^Kursk State University, Kursk, Russia; ^3^Kursk State Medical University, Kursk, Russia; ^4^National Institute for Health Development, Tallinn, Estonia; ^5^Kazan Federal University, Kazan, Russia; ^6^Emanuel Institute of Biochemical Physics, Moscow, Russia

## Abstract

Procedure of manufacturing* K. pinnata* water extracts containing cecropin P1 (CecP1) from the formerly described transgenic plants is established. It included incubation of leaves at +4°C for 7 days, mechanical homogenization of leaves using water as extraction solvent, and heating at +70°C for inactivating plant enzymes. Yield of CecP1 (after heating and sterilizing filtration) was 0.3% of total protein in the extract. The water extract of* K. pinnata* + CecP1 exhibits favorable effect on healing of wounds infected with* S. aureus* (equal to Cefazolin) and with a combination of* S. aureus* with* P. aeruginosa* (better than Cefazolin). Wild-type* K. pinnata* extract exhibited evident microbicide activity against* S. aureus* with* P. aeruginosa* but it was substantially strengthened in* K. pinnata* + CecP1 extract.* K. pinnata* extracts (both wild-type and transgenic) did not exhibit general toxicity and accelerated wound recovery. Due to immunomodulating activity, wild-type* K. pinnata* extract accelerated granulation of the wound bed and marginal epithelialization even better than* K. pinnata* + CecP1 extract. Immunomodulating and microbicide activity of* K. pinnata* synergizes with microbicide activity of CecP1 accelerating elimination of bacteria.

## 1. Introduction

Due to a broad use of antibiotics, drug resistance of microbial pathogens became one of the greatest problems of the modern medicine [1]. It is of a particular importance in complicated chronic conditions, for example, in trophic ulcers in patients with diabetes mellitus where long-term antimicrobial therapy is mandatory [2]. Due to a peculiar mechanism of action, antimicrobial peptides (AMP) are considered to be a promising alternative to the traditional antibiotics [3]. Data about velocity of rising resistance to them in microbial pathogens in comparison to the antibiotics is contradictory since practical application of AMP is much narrower than one of the antibiotics [4]. A high side toxicity and low proteolytic stability often figure as a general reason of a poor AMP (e.g., magainins/bombinins and defensins) applicability in pharmaceutics [5].

AMP cecropin P1 (CecP1) was found for the first time in a swine intestine [6]. Then it was reattributed to a swine helminth* Ascaris suis* abundant in the gut of pigs [7]. In contrast to most other known AMP, CecP1 is naturally adapted to act in a medium with a high level proteolytic activity exhibited by duodenal enzymes. Ubiquitous abundance of* A. suis* in pigs provides an evidence that CecP1 does not impair viability of the mammalian host even when accumulated in a high dosage [8]. Therefore application of CecP1 in pharmacy looks promising. However, for a long time, CecP1 was not available for clinical trials due to absence of an appropriate way for its producing. Solid-phase chemical synthesis is too expensive, liquid-phase synthesis has not been established, and biosynthesis is precluded by a toxicity of CecP1 to potential producers (bacteria, yeast, and micellar fungi). Sophisticated methods of its producing by recombinant strains within fused proteins with decreased toxicity are expensive and difficult for technological implementation [9]. However, there is a broad range of data about microbicide activity of CecP1 towards bacterial and fungal phytopathogens [10] and human/animal pathogens [11]. Virucide and antitumor activity was also attributed to CecP1 [12, 13].

Recently engineering transgenic plants of* Kalanchoe pinnata* expressing CecP1 synthetic gene and accumulating the recombinant AMP in cytoplasm was reported [10]. Bactericide efficiency of pure CecP1 and the wild-type* K. pinnata* extract against model bacterial strains is described [14]. However, data about antimicrobial efficiency of the recombinant CecP1 in vivo and its possible side toxicity are not available. Here we report results of testing medicinal activity of* K. pinnata* extracts containing CecP1 (compared with an extract of a wild-type* K. pinnata*) in a rat model of wounds infected with* Staphylococcus aureus* and* Pseudomonas aeruginosa* strains recently isolated from patients with a purulent infection. Antisuppurative, wound healing, and antimicrobial effect were assessed and used as criteria for comparison of antimicrobial and toxic effects of CecP1 within* K. pinnata* extract in vivo.

*K. pinnata* seems to be a promising bioreactor for CecP1 production since its extract may be used for epicutaneous application without special purification.* K. pinnata* (Lam.) Pers. (syn.* Bryophyllum pinnatum*; family Crassulaceae) is a popular plant used in traditional medicine in many temperate regions of the world and particularly in South America [15]. There are a number of reports about activity of* K. pinnata* extracts against bacteria [16, 17],* Leishmania* [18], insects [19], and even viruses [20, 21].

Wild-type* K. pinnata* extracts harbor a number of bioactive compounds, for example, bufadienolides [22], flavonols (polyphenols and glycosides of syringe acid) [23], and hemagglutinating lectins [24, 25]. They confer complex immunomodulatory [26, 27], lymphoproliferative [18], antioxidant [28, 29], microbicide [30], and cytotoxic [22, 31, 32] properties on the extracts. Some of these effects look directed oppositely. For instance, Da Silva et al. [18] reported activation of Th1-type response (IL-2 and IFN-*γ* overproduction) and suppression of Th2-type response (downregulation of IL-4) by a juice of* Kalanchoe brasiliensis* upon a systemic administration to mice, whereas Umbuzeiro-Valent et al. [33] and El Abdellaoui et al. [22] found an anti-inflammatory (antihistamine) effect in the same preparation. Shirobokov et al. [20] described induction of a blast-transformation in lymphocytes of peripheral blood by a lectin from* Kalanchoe blossfeldiana* likely due to concanavalin A whereas Costa et al. [34] described an antiproliferative effect of patuletin acetylrhamnosides from* Kalanchoe brasiliensis* on human lymphocytes. Taken together, these data do not allow a comprehensive prediction of a synergistic or antagonistic effect of antimicrobial effects of CecP1 and* K. pinnata* juice natural ingredients towards bacterial pathogens in vivo (with involvement of innate immune mechanisms of the animal).

We addressed a problem of producing a highly stable* K. pinnata* extract bearing CecP1 and testing its therapeutic activity towards* Staphylococcus aureus* purulent mono infection and its combination with* Pseudomonas aeruginosa* (as an example of typical nosocomial infection). The experiment was carried out in a model of infected planar wounds in rats. Experimental samples of* K. pinnata* extracts bearing CecP1 were compared with a wild-type* K. pinnata* extract and with a commonly used antibiotic Cefazolin (first-generation semisynthetic cephalosporin with a predominant activity towards Gram-positive bacteria) [35]. A subgroup of nontreated rats infected with the same bacterial pathogens was used as a negative control. The therapeutic effect was estimated by wound healing activity (planimetric method) and by microbicide activity (microbiological study of tissue samples in the wound cavity).

## 2. Methods

### 2.1. Producing* K. pinnata* Extracts

*K. pinnata* extracts were prepared from leaves of* K. pinnata* transgenic plants bearing a binary vector for* Agrobacterium tumefaciens* vector with T-element randomly integrated to a plant genome. The vector did not contain a drug resistance marker. The plants selected by a direct immunological testing exhibited a highly stable yield of CecP1 for at least two years as proved by immunoblotting, antimicrobial plate test, and HPLC combined with mass-spectroscopy detection. 5.07 L of extract was produced from 3 kg of recombinant plant leaves using deionized water as an extracting solvent. After sterilizing filtration through nylon membrane with 0.22 *μ*m pores the extract contained 1 mg/mL total protein and 0.7 *μ*g/mL CecP1. The extract of wild-type* K. pinnata* was produced by the same method and adjusted to the same concentration of the total protein. Synthetic CecP1 peptide described previously [14] was used as a standard for quantification of CecP1 in the recombinant* K. pinnata* extracts.

### 2.2. Animals

This experimental study in vivo was organized according to European Convention about defense of the vertebrates used for experiments or for another scientific aims (Strasbourg, Mar 18, 1986) of ETS N123. 240 adult male Wistar rats weighing 180 ± 20.0 g and aged 3-4 months were totally allocated for the experiment. After quarantine they were placed into individual cages. All animals were contained in equal terms on a standard diet: twelve hours of darkness and 12 hours of light were available. They had a ready access to water and food.

The animals were randomly divided into groups (60 animal each) treated with wild-type* K. pinnata* extract, recombinant* K. pinnata* extract containing CecP1 (*K. pinnata* + CecP1), Cefazolin (positive control), respectively, and a negative control group (mock treatment with a saline). Each group was randomly divided into two subgroups (30 animals each) infected with (1)* S. aureus* and (2)* S. aureus* +* P. aeruginosa*. Each subgroup was divided into three echelons withdrawn from the experiment at 3rd, 10th, and, 14th day after beginning of curing.

### 2.3. Bacterial Strains

*Erwinia carotovora* subsp.* carotovora* ATCC 15713 (type strain) was cultivated at plates with LB medium (peptone bacto (Difco) 10 g/L, yeast extract (Difco) 5 g/L, NaCl 10 g/L, and agar 15 g/L) at 25°C.

The bacterial pathogens* Staphylococcus aureus* (ATCC 25923) and* Pseudomonas aeruginosa* (ATCC 27853) isolated from clinical specimens were purchased from type strain collection of Tarasevich Research Institute for Standardization and Control of Medical Biological Preparations. The strains were grown for 18–20 h at slant IPA (meat-peptone nutrient agar) supplemented with 0.1% glucose. The fresh cultures were rinsed out with a sterile saline, thoroughly suspended, adjusted to a concentration ~10^9^ CFU per mL by using an optical turbidity standard CCA 42-28-29-85, and used for inoculation of wounds.

### 2.4. Surgical Manipulations, Treatment, and Planimetry Assay of the Wounds

A purulent infection was modeled in rat using a method described previously [36]. The animals were anesthetized with ether. A square shape 20 × 20 cm skin area at the animal back was thoroughly shaved and treated by a disinfectant (70% ethanol); derma and epidermis were surgically removed. 1 mL of bacterial suspension containing 10^9^ CFU/mL* S. aureus* ATCC 25923 or 10^9^ CFU/mL* S. aureus* ATCC 25923 and 10^9^ CFU/mL* P. aeruginosa* ATCC 27853 was distributed at the surface of the wound. For standardizing the wound healing conditions the wound cavity was closed with a gauze bandage coupled to the skin.

36 h after wounding and infection all animals exhibited clear symptoms of suppuration and inflammation. In this moment the stitches and the bandage were removed, and the wound cavity was thoroughly washed from the pus. The wound area was determined by its lineation at a sterile transparent film. Then the wounds were treated with 3% hydrogen peroxide and subjected to a specific treatment. The described wound treatment was repeated daily for 14 days after beginning of the curing.

The wounds in the control subgroup were treated with 3% hydrogen peroxide and with a sterile saline. Other subgroups instead of the saline were treated with 10% Cefazolin or with undiluted* K. pinnata* extract containing 1 mg/mL total protein (experimental preparation contained 0.7 *μ*g/mL CecP1).

The animals were examined daily and stages of wound healing (inflammation, granulation, and maturation (marginal epithelialization)) were fixed.

The planimetric analysis of the wound recovery percentage was carried out at 3rd, 10th, and 14th day after beginning of curing. After this one echelon (10 animals from each subgroup) was withdrawn from the experiment. The animals were sacrificed with overdose of the ether anesthesia.

Wounds on different days were measured as described formerly [37] by transparent sheet which was scanned at resolution 200 pcs/inch. The image was acquired in a format Adobe Photoshop CS5 Extended. The object was selected and its square was automatically calculated by selecting a menu command “Analysis.” An average mean and a standard error (M ± Std.  Err.) were calculated. The recovery percentage was evaluated with following formula:

recovery percentage = (wound surface on the day zero of curing − wound surface on day *X*)/wound surface on the day zero of curing × 100, where *X* = day of wound surface measurement.

### 2.5. Bacteriological Analysis of Microbial Load in Wound Cavity Tissue

0,1–0,5 g tissue (fibrous mass, infiltrate, and underlying derma) was sampled from a sacrificed animal under aseptic conditions, weighed at analytical grade balances (accuracy 0.1 mg), placed in a sterile porcelain mortar, mixed with a sterile saline in a ratio 1 : 10, and homogenized with a sterile pestle for 3 min. The homogenate was diluted 1000 times with a sterile saline (with three consequent steps 1 : 10 using 1 mL samples) and 100 *μ*L aliquot of each dilution was inoculated to Petri dishes with IPA (meat-peptone nutrient agar) supplemented with 0.1% glucose. The inoculated dishes were incubated at 37 ± 1°С for 20 h and then 1 day more at a room temperature. The colonies were counted and number of CFU recalculated per 1 g tissue. The count was suggested to be valid if number of colonies was between 30 and 300.

### 2.6. Statistical Analysis

Statistical analysis of research results was performed by Microsoft Excel 2007 and program “Statistics” 8.0 StatSoft. The averages of quantitative indexes and standard errors of mean were calculated. Authenticity of distinctions of averages between the series of comparison and other series was estimated by the Mann–Whitney *U* test (*p* < 0.05).

## 3. Results and Discussion

### 3.1. Producing* K. pinnata* Extracts

**Figure 1:**
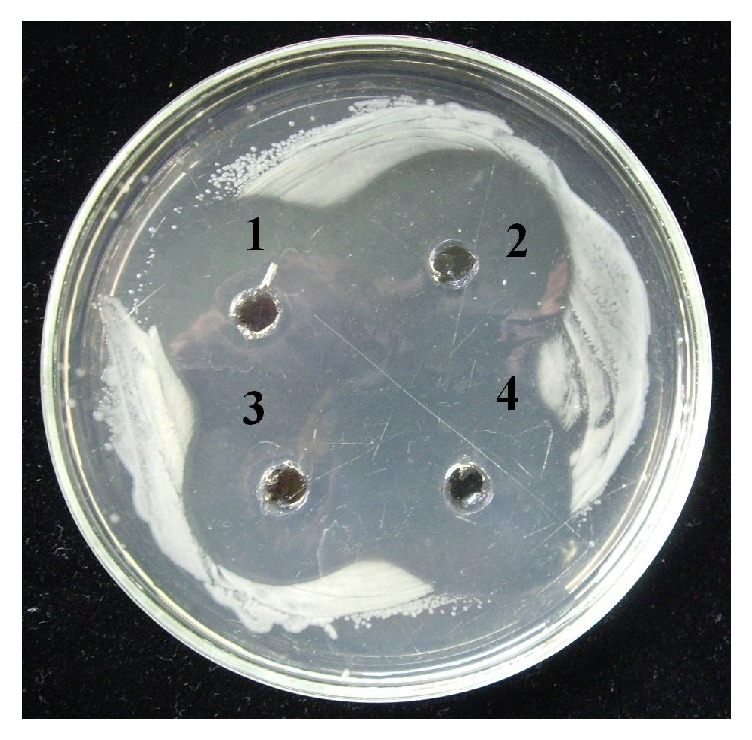
Agar diffusion test for determination of antibacterial activity towards* E. carotovora* in extracts of* K. pinnata* transgenic lines. (1)* K. pinnata*, line #1, halo 29 mm; (2) synthetic CecP1 (1 *μ*g/mL), halo 34 mm; (3)* K. pinnata*, line #2, halo 26 mm; (4)* K. pinnata* line #3, halo 28 mm.

Three independent* K. pinnata* transgenic lines (~1-year-old plants height 2–2,5 m) and a wild-type parental* K. pinnata* of the same age cultivated in a green-house under a constant light/darkness and temperature regime were used for producing the extracts. Middle tier leaves were used for preliminary testing of CecP1 yield. 200–300 mg specimens were sampled, placed in Eppendorf tubes, and weighed and mixed with NEB buffer (10% glycerol, 4 mM EDTA, 150 mM NaCl, 100 mM NH_4_Cl, 10 mM Tris-HCl, pH 7.5, 0.2 mg/mL leupeptin, and 0.2 mg/mL trypsin inhibitor) in ratio 20 *μ*L per 100 mg leaf tissue. The leaf specimens were homogenized with a glass stick in the Eppendorf tubes and total protein was measured by Bradford method (Sigma-Aldrich B6916 ready-to-use kit was used following instructions of manufacturer). The extracts were adjusted to the total protein concentration 1 mg/mL with NEB buffer and used for determination of antimicrobial activity by diffusion in agar against* E. carotovora* subsp.* сarotovora*. 10^8^ CFU/plate were distributed at 90 mm Petri dishes with LB medium using top agarose method. Wells with diameter 5 mm were pierced in the agar and 100 *μ*L aliquots of the plant extracts were dripped to each well. A synthetic CecP1 (1 *μ*g/mL) in amount 100 *μ*L per well was used as a standard. Results of the testing are shown at Figure 1.

Data of Figure 1 allow estimating yield of CecP1 contents in the leaves of* K. pinnata* transgenic lines ~0.3–0.5 of the total soluble protein. Transgenic line (1) was chosen for preparative manufacturing of the extract. An extract of the wild-type parental* K. pinnata* was prepared in parallel. 3.0 kg cut leaves were incubated at +4°С for 7 days in darkness. The leaves were mixed with the ice-cold water (1.0 L water per 1 kg leaves) and homogenized with a hand electric blender. The homogenate was dark-green. It was clarified by centrifugation at 6000*g* for 30 min. The green pallet was discarded. The straw-yellow extract was placed in 0.5 L glass flasks and incubated at +70°C in a water bath for inactivating enzymes. The extract was clarified by centrifugation at 6000*g* for 30 min and sterilized by filtration through a 0.22 *μ*m filtering cartridge (Hydrophilic PNN Membrane Filter, Hangzhou Kosma Membrane Technology Co., PRC) under aseptic conditions. The extracts were stored at +4°C in culture flasks of nominal volume 0.5 L hermetically closed with rubber plugs and aluminum caps until used for biological testing.

**Figure 2:**
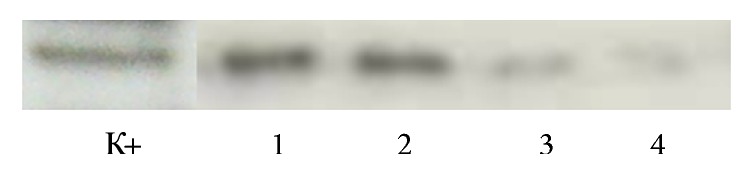
Immunoblotting analysis of* K. pinnata* extracts. (*К*+) synthetic CecР1, Mr = 3.4 kDa (30 ng); (1)* K. pinnata* transgenic line 1 after the cold stress; (2)* K. pinnata* transgenic line 2 after the cold stress; (3)* K. pinnata* transgenic line 2 before the cold stress; (4)* K. pinnata* transgenic line 2 before the cold stress.

Presence of CecP1 in the extracts was tested using immunoblotting as described formerly [10] (Figure 2). Briefly, 10.0 *μ*L of extract and 30.0 *μ*L of the control synthetic CecP1 (1 *μ*g/mL) were pelleted with 10% trichloroacetic acid, denatured and separated in 15% polyacrylamide gel, blotted to nitrocellulose membrane, and stained with rabbit polyclonal antibodies.

The immunoblotting demonstrated upregulation of CecP1 expression in* K. pinnata* leaves under cold stress conditions. Further antimicrobial activity of the extracts was confirmed in the agar diffusion test at* E. carotovora* model as described above. Overall report about manufacturing* K. pinnata* extracts containing CecP1 is shown in Table 1.

### 3.2. Testing Healing of the Wounds Infected with* S. aureus*

**Figure 3:**
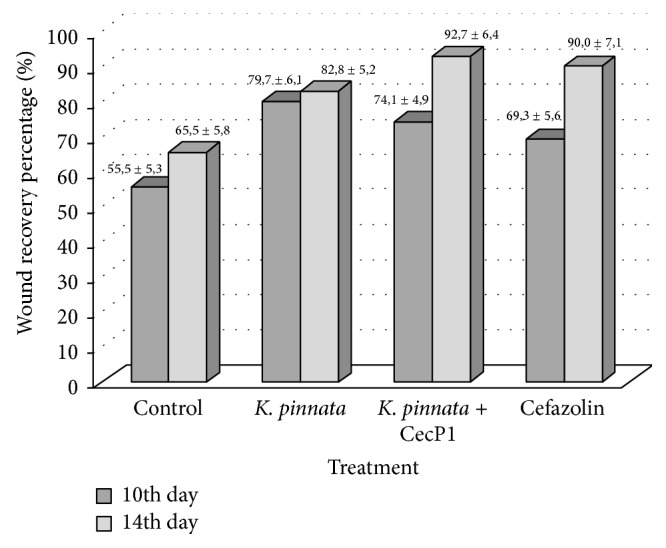
Testing healing of the wounds infected with* S. aureus.* Wound recovery percentage at 10th and 14th days after beginning of curing (M ± Std.  Err.) is shown.

*S. aureus* is the most common causative agent of the nosocomial purulent infections. It is prone to raising multiple drug resistance. As found in 3-month epidemiological study to determine the prevalence and antibiotic resistance of* S. aureus* nosocomial infections in 52 centers throughout Italy in 2012, the prevalence of* S. aureus* among all nosocomial pathogens isolated in that period was 11.6% (*n* = 2541), whilst the prevalence of methicillin-resistant* S. aureus* (MRSA) among the* S. aureus* was 35.8% (*n* = 910) [38]. However, the collection* S. aureus* strain ATCC 25923 exhibited a high susceptibility to Cefazolin in vitro and was chosen as a model object for in vivo studies of efficiency of* K. pinnata* extracts which was compared with this antibiotic. Data at Figure 3 demonstrate that the wound recovery percentage is significantly higher in* K. pinnata* + CecP1 subgroup than in the control subgroup (mock treatment): the difference is characterized with the third threshold of validity (*p* < 0.001) at 10th and 14th days. The final wound healing activity of* K. pinnata* + CecP1 did not differ from one of Cefazolin groups: *p* > 0.05 at days 10 and 14 when two subgroups were compared. Wild-type* K. pinnata* extract exhibited a certain efficiency (difference with the negative control was characterized with the first threshold of significance at 10th and 14th days after beginning of curing). However, the wound healing activity of* K. pinnata* + CecP1 extract was significantly higher than in the wild-type* K. pinnata* extract (*p* < 0.01 at 10th day and *p* < 0.05 at 14th day). This gives an evidence of a clear CecP1 contribution to the own* K. pinnata* extract therapeutic efficiency.

**Figure 4:**
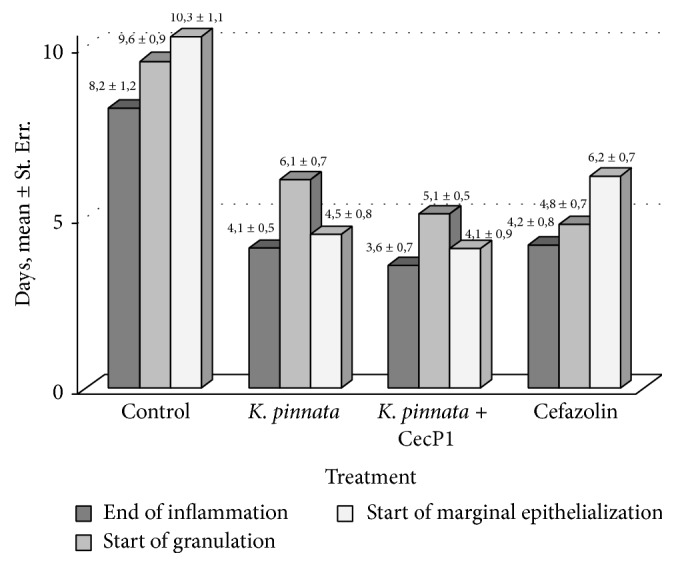
Testing healing of the wounds infected with* S. aureus.* Timing of wound recovery phases after beginning of curing (M ± Std.  Err.) is shown.

Positive results were obtained when timing of wound recovery phases was compared between the animal subgroups. Data of Figure 4 demonstrate that the inflammation phase ends about 2 times faster in all three experimental subgroups than in the control subgroup without specific treatment. However, start of granulation phase in* K. pinnata* subgroup (but not in* K. pinnata* + CecP1) was found about 1 day earlier than in Cefazolin subgroup (difference between the subgroups first threshold is characterized with the first threshold of significance, *p* < 0.05). Marginal epithelialization (maturation phase) was found almost 2 days faster in* K. pinnata* and* K. pinnata* + CecP1 subgroups than in Cefazolin subgroup (*p* < 0.01). Taken together these data prove a favorable effect of* K. pinnata* extract components for healing of the infected wounds which cannot be substituted with antibiotic and strengthening the antimicrobial activity.

### 3.3. Testing Healing of Wound Infected with a Combination of* S. aureus* and* P. aeruginosa*

Mixed purulent infections usually are heavier and difficult for curing than mono infections. Men'shikov et al. [39] reported that complex infection of* S. aureus* and* P. aeruginosa* is associated with the most unfavorable prognosis in patients with burns. In contrast to* S. aureus, P. aeruginosa* is usually resistant to Cefazolin [35]; therefore outcome of treatment of a mixed infection of* S. aureus* and* P. aeruginosa* is poorly predictable.

**Figure 5:**
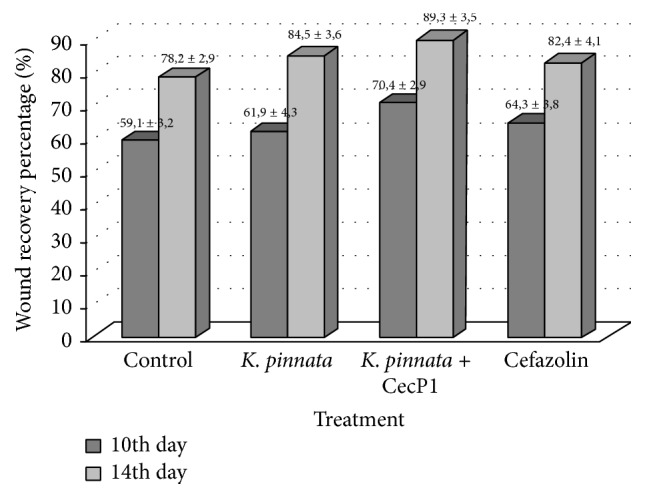
Testing healing of the wounds infected with combination of* S. aureus* and* P. aeruginosa.* Wound recovery percentage at 10th and 14th days after beginning of curing (M ± Std.  Err.) is shown.

Experimental study of wounds infected with equal doses of* S. aureus* and* P. aeruginosa* demonstrated that their recovery in the control subgroups (without treatment) does not differ significantly from* S. aureus* mono infection and even goes somewhat faster (Figure 5). However, treatment of* S. aureus* +* P. aeruginosa* with Cefazolin is significantly less efficient than* S. aureus* mono infection (*p* < 0.01). Comparison of the control and Cefazolin subgroups within the mixed infection group demonstrated no significant difference (*p* > 0.05). Therefore, Cefazolin can be acknowledged to be inefficient for treatment of the mixed purulent infection.

Using the wild-type* K. pinnata* extract gives somewhat better results than Cefazolin for curing the mixed infection. Difference between* K. pinnata* and the control subgroups is characterized with *p* < 0.05 for 14th day (*p* > 0.05 for 10th day). At this background, efficiency of* K. pinnata* + CecP1 extract with the control looks highly promising: *p* < 0.01 for 10th day and *p* < 0.001 for 14th day after beginning of curing.

**Figure 6:**
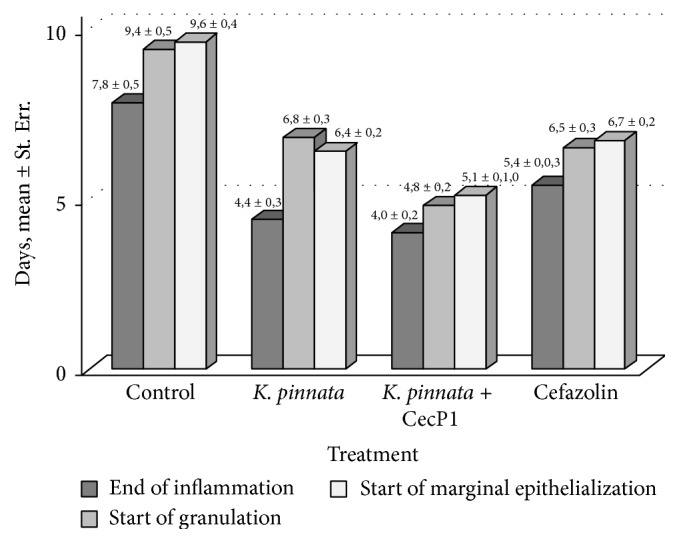
Testing healing of the wounds infected with combination of* S. aureus* and* P. aeruginosa.* Timing of wound recovery phases after beginning of curing (M ± Std.  Err.) is shown.

When timing of the wound healing was compared in the mixed infection group of animals, Cefazolin was found to be much more efficient than for the wound recovery percentage (Figure 6). Difference between Cefazolin and the control subgroups was characterized with *p* < 0.001 for all three parameters (end of inflammation, start of granulation, and start of marginal epithelialization).

On the other hand, comparison of* K. pinnata* and* P. pinnata* + CecP1 subgroups demonstrated no difference in timing of end of inflammation (*p* > 0.05), whereas granulation and marginal epithelialization were found to start earlier in* K. pinnata* subgroup. This ambiguous result cannot be explained by effect of CecP1 only. Apparently, immunomodulating effect of own* K. pinnata* biologically active compounds (flavonoids or lectins) for granulation and epithelialization was more pronounced than antimicrobial; effect of CecP1 and production of these compounds was higher in the control* K. pinnata* plants than on the transgenic plants expressing CecP1. In turn, this hypothesis is not in a good agreement with an evident impact of Cefazolin on the timing of the wound healing phases but not on the wound recovery rate.

### 3.4. Testing Microbicide Activity of* K. pinnata* Extracts In Vivo

Planimetrical studies of wound healing provide key information about therapeutically efficiency of the antimicrobial means. However, they must be supplemented with direct data about survival of the microbial agents in the wound bed. These data are important for optimization of the treatment scheme.

**Figure 7:**
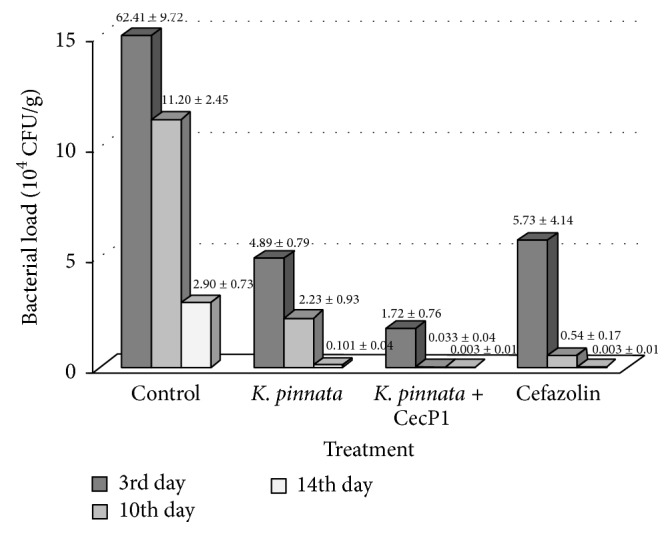
Testing microbicide activity of* K. pinnata* extracts against* S. aureus* mono infection in vivo.

Data at Figure 7 demonstrate collapsing bacterial load of* S. aureus* along with the wound healing. This phenomenon is evident even in the control subgroup (without treatment); however it is much greater in the experimental subgroups. On the other hand, residual bacteria are found in all subgroups even at 14th day of the experiment. All four subgroups exhibit statistically significant differences between each other in the bacterial load at 3rd and 10th day of the experiment (*p* < 0.01) following in the order: control > Cefazolin >* K. pinnata* >* K. pinnata* + CecP1. In contrast, no valid differences between the subgroups in the bacterial load are found at 14th days after beginning of curing. This observation proves existence of refugees in vivo where the pathogen can take a cover even against drugs with high microbicide potency. This effect may contribute to rising drug resistance in bacteria by providing time necessary to the pathogen for adaptation to the therapy.

**Figure 8:**
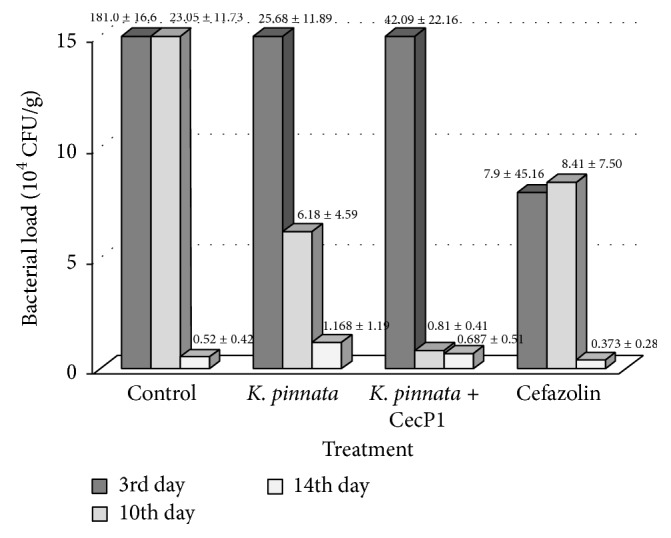
Testing microbicide activity of* K. pinnata* extracts against combination of* S. aureus* and* P. aeruginosa* in vivo.

Picture of the microbicide activity of* K. pinnata* extracts against combination of* S. aureus* and* P. aeruginosa* roughly repeat the picture of the microbicide activity against* S. aureus* mono infection (Figure 8). There is a statistical significance of the bacterial load between all groups (*p* < 0.01) at 10th (not 3rd) day of experiment. Microbicide effect of Cefazolin against the mixed infection was weak but not zero. Extract of the wild-type* K. pinnata* was less efficient than* K. pinnata* + CecP1 (*p* < 0.05) at 10th and 14th days of experiment. However, residual bacterial load at 14th day of the experiment was much higher in the mixed infection than in* S. aureus* mono infection.

## 4. Conclusions

Transgenic line of* K. pinnata* with the highest CecP1 was selected 1 year after its obtaining. A cold stress induction of CecP1 synthesis in cut* K. pinnata* leaves incubated at +4°C for 7 days allowed increased yield of CecP1 1.5–2 times. A simple and efficient procedure of water extraction CecP1 from* K. pinnata* leaves was established. In contrast to the commonly used ethanol extraction [21, 22], it allowed preservation of hemagglutinating lectins described in* Kalanchoe* [25] and exhibiting lymphoproliferative activity [20]. However, these proteins may be lost due to denaturation in the course of the extract heating at +70°C. Heating the extract was mandatory for inactivation of proteases which otherwise degraded CecP1 under the storage for 7 days at +4°C.

Following our data, the extracts of* K. pinnata* (wild and transgenic) did not exhibit any cytotoxic activity towards the wound bed or general toxicity at animals in the applied concentration (1 mL of the extract per dose, total protein concentration 1 mg/mL) although such toxicity was previously ascribed to both* K. pinnata* extracts [19] and synthetic CecP1 [3]. Water extract of wild-type* K. pinnata* exhibited both microbicide and wound healing activity, although its own microbicide activity was lower than in Cefazolin. In contrast,* K. pinnata* + CecP1 extract exhibited the same microbicide activity against* S. aureus* mono infection as Cefazolin. Activity of* K. pinnata* + CecP1 extract against combination of* S. aureus* and* P. aeruginosa* was much better than in Cefazolin.

Noteworthily, none of the examined preparations did provide complete elimination of bacteria in the wound bed even on the 14th day of the experiment. Residual bacterial load at this period was close for all three microbicides. This effect, despite a low number of the residual alive bacteria, may contribute to rising drug resistance in pathogens and should be controlled.

Taken together, the extract of* K. pinnata* transgenic plants containing 0.7 *μ*g/mL CecP1 obtained following the described method may be suggested to be promising for external use. First of all, it should be tested as a candidate drug for treatment of trophic ulceration in patients with diabetes mellitus.

## Figures and Tables

**Table 1:** Reproducibility of manufacturing *K. pinnata* extracts containing CecP1.

Period of operation	*K. pinnata *leaves, kg	Total yield of CecP1, mg
Mar 15, 2016–Mar 29, 2016	3.04	3.63
Mar 30, 2016–Apr 5, 2016	3.02	3.45
Apr 6, 2016–Apr 20, 2016	3.04	3.34
